# Cut and paste: temperature-enhanced cotyledon micrografting for *Arabidopsis thaliana* seedlings

**DOI:** 10.1186/s13007-020-0562-1

**Published:** 2020-02-05

**Authors:** Kai Bartusch, Jana Trenner, Charles W. Melnyk, Marcel Quint

**Affiliations:** 1 Institute of Agricultural and Nutritional Sciences, Martin Luther University Halle-Wittenberg, Betty-Heimann-Str. 5, 06120 Halle (Saale), Germany; 2 Institute of Computer Science, Martin Luther University Halle-Wittenberg, Von-Seckendorff-Platz 1, 06120 Halle (Saale), Germany; 3 Department of Plant Biology, Swedish University of Agricultural Sciences, Ulls gränd 1, 765 51 Uppsala, Sweden

**Keywords:** Cotyledon, Grafting, Cotyledon micrografting, Cot-grafting, Petioles, Temperature, Arabidopsis

## Abstract

**Background:**

Cotyledon micrografting represents a useful tool for studying the central role of cotyledons during early plant development, especially their interplay with other plant organs with regard to long distance transport. While hypocotyl micrografting methods are well-established, cotyledon micrografting is still inefficient. By optimizing cotyledon micrografting, we aim for higher success rates and increased throughput in the model species *Arabidopsis thaliana*.

**Results:**

We established a cut and paste cotyledon surgery procedure on a flat and solid but moist surface which improved handling of small seedlings. By applying a specific cutting and joining pattern, throughput was increased up to 40 seedlings per hour. The combination of short-day photoperiods and low light intensities for germination and long days plus high light intensities, elevated temperature and vertical plate positioning after grafting significantly increased ‘ligation’ efficiency. In particular high temperatures affected success rates favorably. Altogether, we achieved up to 92% grafting success in *A. thaliana*. Reconnection of vasculature was demonstrated by transport of a vasculature-specific dye across the grafting site. Phloem and xylem reconnection were completed 3–4 and 4–6 days after grafting, respectively, in a temperature-dependent manner. We observed that plants with grafted cotyledons match plants with intact cotyledons in biomass production and rosette development.

**Conclusions:**

This cut and paste cotyledon-to-petiole micrografting protocol simplifies the handling of plant seedlings in surgery, increases the number of grafted plants per hour and greatly improves success rates for *A. thaliana* seedlings. The developed cotyledon micrografting method is also suitable for other plant species of comparable size.

## Background

Since ancient times, plant grafting has been successfully applied to various plant species for horticultural and agricultural purposes [1–3]. Recently, grafting of small plant seedlings, often termed micrografting, has become an attractive tool to investigate physiological responses which depend on organ-to-organ long-distance transport of various substances [4]. For example, small transported molecules like plant hormones [5–9], small RNAs [10, 11], small peptides/proteins [12, 13], nutrients [14, 15], glucosinolates [16], or phytochelatins [17] have been studied using micrografting.

Grafting in *Arabidopsis thaliana* was described over 20 years ago [18, 19] and has since then been applied to different organs, including inflorescences [20], cotyledons [13] and shoots/roots [5, 19]. While inflorescence stem grafting and hypocotyl micrografting have reported success rates of up to 87% [20] and 100% [21, 22], respectively, cotyledon micrografting (cot-grafting) success rates have been very low so far (< 2% [13]). Despite this constraint, cot-grafting was already successfully applied to reveal the important role of cotyledons in floral development [13, 23]. Mobile FLOWERING LOCUS T (FT) protein, for example, is produced in significant amounts in the cotyledons and transported via the phloem to the shoot apical meristem, where FT induces floral transition.

Although the role of cotyledons in plant development and their interaction with other plant organs is of great importance [24–26], cot-grafting has not been utilized for other approaches probably because of the low success rate as illustrated by the only described method so far [13]. Due to the miniature size of the seedlings and their cotyledons, cot-grafting is a technically challenging process in *A. thaliana* and other small plant species. Frequently, the cotyledons do not remain attached to the petioles of the recipient plants, possibly caused by the circumnutation of cotyledons driven by the circadian clock [13, 27]. This may be one of the main reasons for the low success rate.

As shoot–root hypocotyl micrografting in *A. thaliana* has become a well-established method in fundamental plant research [4], we aim to provide an optimized cot-grafting method to encourage further research of cotyledons and their important role in plant growth and development. Therefore, we developed a flat-surface cotyledon-to-petiole micrografting protocol which simplifies handling of grafted seedlings, increases the number of cot-grafted plants per hour, and produces high success rates (up to 92%) for young *A. thaliana* seedlings.

## Methods

We developed cut and paste cot-grafting performed on a flat solid surface based on the general conditions for hypocotyl micrografting [22]. The grafting procedure was then adjusted for cotyledon transplantation. Furthermore, the impact of different growth conditions on cot-grafting success rates was evaluated before and after grafting.

### Plant material

Micrografting experiments were performed with wild type *A. thaliana* seedlings (Col-0, obtained from the INRA collection as AV186), unless indicated otherwise. For phloem formation assays, pSUC2::GFP (Col-0 background) [28] was used. Using the growth conditions with the highest success rate (Table 1) for *A. thaliana*, cot-grafting was also investigated in *Capsella rubella* (NASC ID N22697), *Arabidopsis suecica* (NASC ID N22505), *Brassica napus* (NASC ID N29003) and *Solanum lycopersicum* L. cv. Castlemart.

**Table 1:**
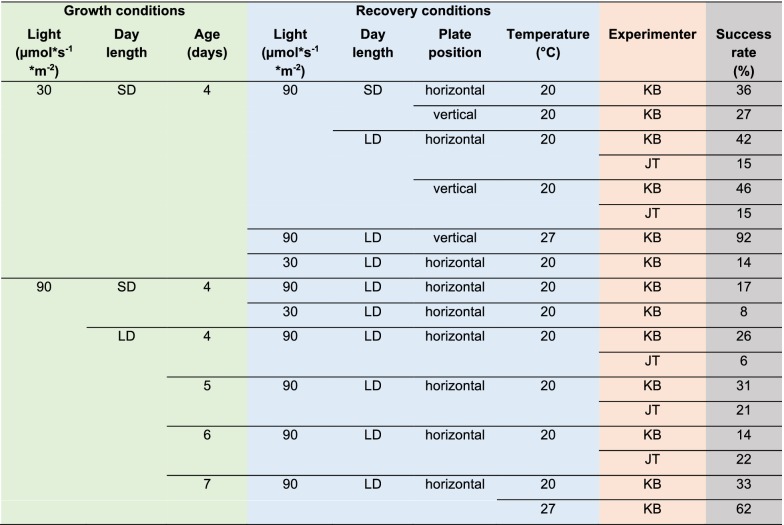
Growth conditions before cot-grafting and the recovery conditions thereafter affect the graft union

Seedlings of *Arabidopsis thaliana* were germinated at different light intensities, day length and grafted at different ages. After grafting, different light intensities, day length, plate positions, and temperature levels were tested as recovery conditions. The experimenters KB and JT are indicated to evaluate human impact. 72 plant seedlings were grafted per combination and the graft union was evaluated 7 days after surgery

SD, short day conditions (8 h of light and 16 h of darkness); LD, long day conditions (16 h of light and 8 h of darkness)

### Growth conditions before cot-grafting

In general, sterile conditions are recommended for micrografting [13, 21, 22]. Seeds were sterilized using a standard protocol [29] and were stored at 4 °C for at least 7 days for stratification. Seeds were sown onto *A. thaliana* solution medium (ATS) [30] without sucrose under a laminar flow hood. The plants were grown in a growth cabinet (Conviron Adaptis A1000) under different light and day length settings (Table 1): Long day (16 h light, 8 h darkness) and short day (8 h light, 16 h darkness) were applied as two different day lengths. The light intensity was 30 µmol * s^−1^ * m^−2^ or 90 µmol * s^−1^ * m^−2^ (T5 white fluorescence lamps with 4000 K). Growth temperatures before grafting were always constant 20 °C. The impact of the seedling’s age on cot-grafting was investigated using 4-, 5-, 6- and 7-day-old plants.

### Cot-grafting procedure

**Fig. 1:**
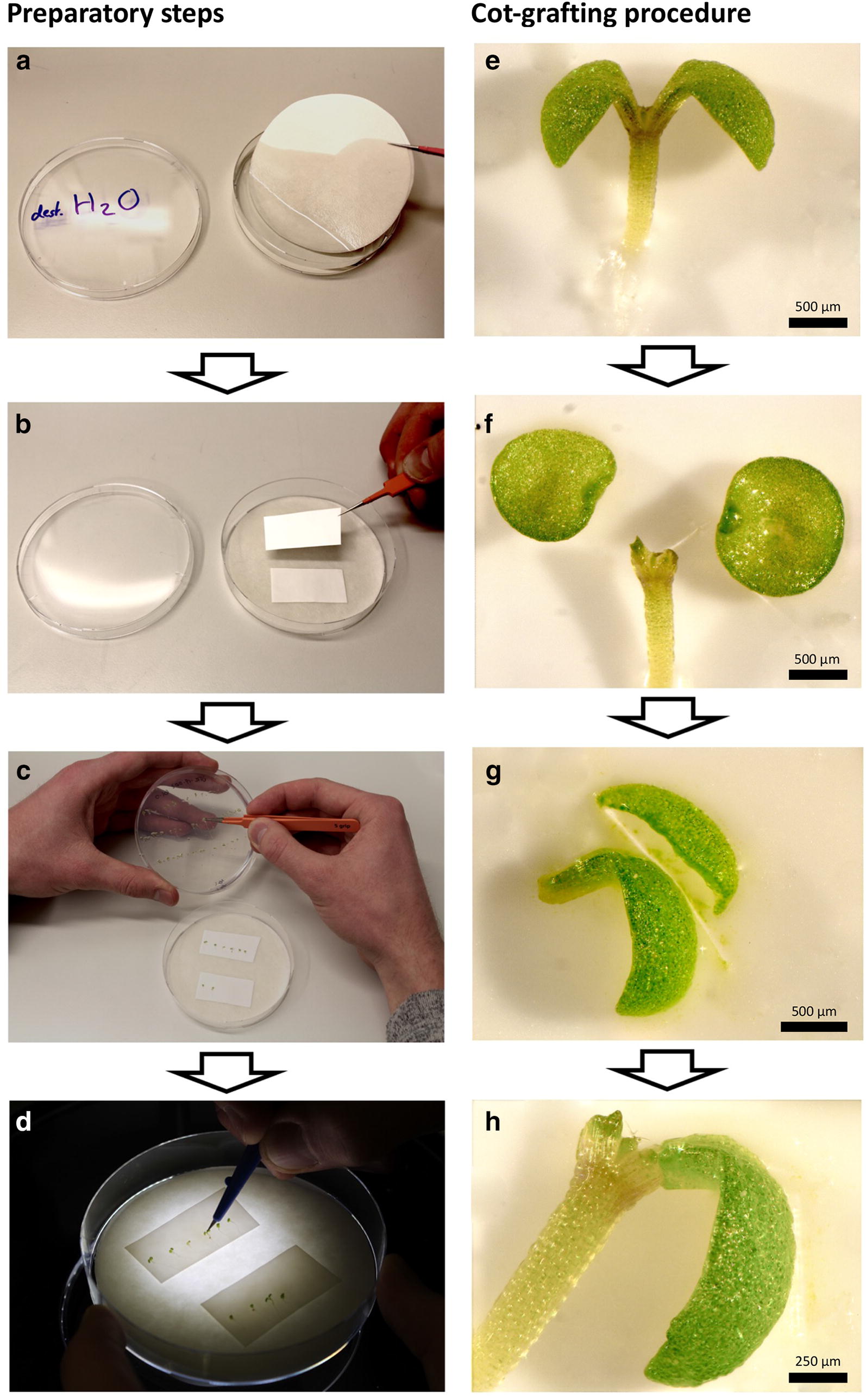
Workflow of cotyledon micrografting (cot-grafting) preparation and procedure. **a**–**d** Preparatory steps: Two layers of sterile filter paper were moistened with distilled water and placed into a sterile petri dish (**a**). Two stripes of nylon membrane were positioned on top of the filter papers (**b**). Vigorous seedlings were selected and placed onto the membrane using fine forceps (**c**). Cot-grafting was performed using a micro knife and a dissecting microscope (**d**). **e**–**h** Cot-grafting procedure: The cotyledons of donor and recipient plants were cut off (**e**, **f**). The donor cotyledon was cut alongside the central leaf vein on one side for optimal positioning (**g**). The donor cotyledon was finally transferred to the recipient plant (**h**). Scale bars correspond to 500 μm (**e**–**g**) and 250 μm (**h**), respectively

**Fig. 2:**
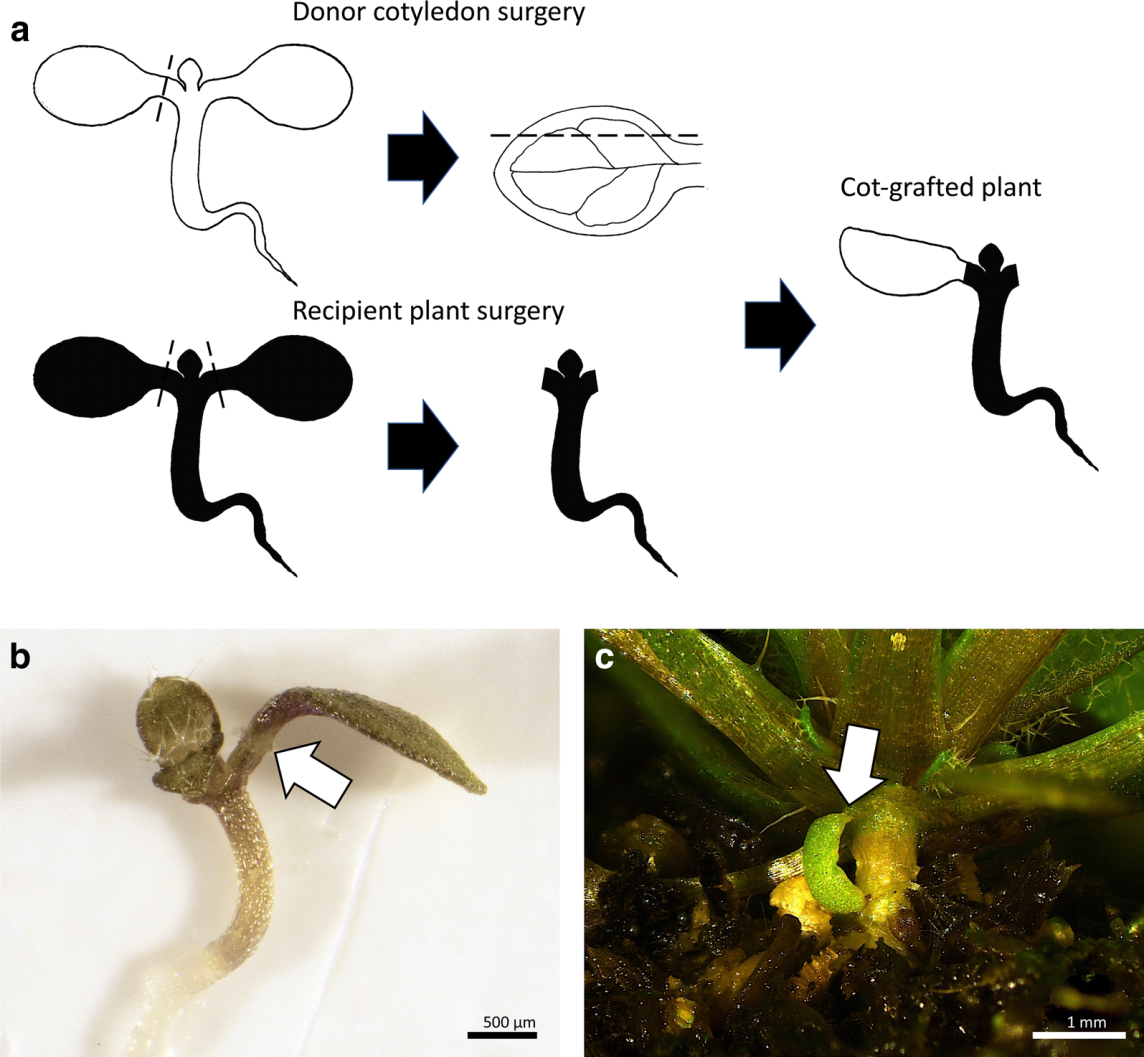
Cut and paste cotyledon micrografting (cot-grafting). **a** The general procedure of cotyledon micrografting: first, one cotyledon was cut from a donor plant. A second cut alongside the central leaf vein created a flat contact surface for optimal positioning (donor cotyledon surgery). Both cotyledons of the recipient plant were cut off (recipient plant surgery). The donor cotyledon was then transplanted to the recipient plant. Black dashed lines indicate cutting edges. A successfully grafted plant 7 days (**b**) and 31 days after surgery (**c**). The arrows mark graft junctions. Scale bars correspond to 500 μm (**b**) and 1 mm (**c**), respectively

The general micrografting environment described in detail by Melnyk [22] was implemented with some modifications: Although plant surgery is recommended to be carried out under a laminar flow, we worked on a lab bench under usual lab conditions. The dissecting tools—fine forceps and Ultra Fine Micro Knives (Fine Science Tools)—were sterilized with ethanol (70%). All other materials were autoclaved before use, if possible. A Motic SMZ168 and a Zeiss Stemi DV 4 were used as dissecting microscopes. The general workflow of the developed cot-grafting method and our established cutting patterns are illustrated in Figs. 1 and 2a. Two round filter papers were moistened with distilled water and were placed into a sterile petri dish (Fig. 1a). An excess of liquid was removed. Two strips of Hybond N membrane were positioned on top of the filter papers (Fig. 1b). Additional moistened strips of filter paper were used to maintain an optimal level of humidity during the micrografting procedure. The recipient seedlings were transferred from the culture media plates to the nylon membrane using fine forceps (Fig. 1c) and placed as flat as possible onto the membrane surface. A slight rotation of the hypocotyl could help to level out the seedling. Throughout the cot-grafting procedure, it was very important to handle the seedlings carefully in order to prevent tissue damage. After these preparatory steps, the microsurgery was conducted under the dissecting microscope (Fig. 1d). Both cotyledons of the recipient plant were removed by precise cuts using a micro knife (recipient plant surgery; Figs. 1f, 2a). Next, the cotyledons of the donor plant were cut off. A longitudinal cut along the central leaf vein was performed preparing the right angle between the cotyledon and the petiole. This step is essential for a natural orientation of the donor cotyledon to the recipient plant (donor cotyledon surgery; Figs. 1g, 2a). The donor cotyledon was then transplanted upright to the recipient petiole using forceps. If the cutting edges at the petioles did not fit well, a small piece of the petiole was cut off to obtain fitting angles between the petioles of the donor cotyledon and recipient plant. After surgery, the petri dishes were sealed using Parafilm^®^ M.

### Growth conditions after cot-grafting

Cot-grafted plants were transferred back to the growth cabinet at 20 °C or 27 °C. In order to investigate recovery conditions after surgery (Table 1), the petri dishes were placed vertically or horizontally. The light intensity was 30 µmol * s^−1^ * m^−2^ or 90 µmol * s^−1^ * m^−2^ and day length was set to long or short day conditions.

### Evaluating the cot-grafting success

The transplanted cotyledons were evaluated 7 days after surgery. To test whether a grafted cotyledons was detachable, fine forceps were used to move it gently. Non-detachable, and therefore well-attached cotyledons were counted as successful graftings. To evaluate human impact on cot-grafting success, we also distinguished between the experimenters KB and JT.

### Vasculature formation and connectivity

**Fig. 3:**
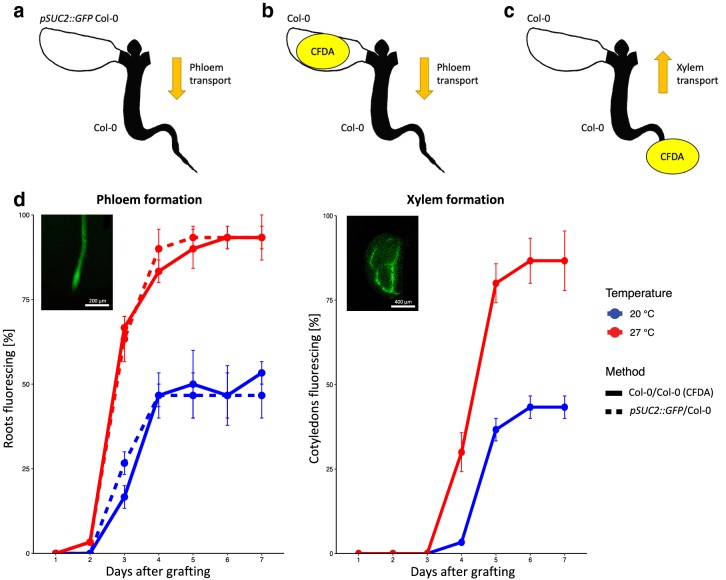
Phloem and xylem formation occur temporally separated. **a**–**c** Cartoons showing the vascular transport assays used. **a***pSUC2::GFP* Col-0 cotyledons were grafted to Col-0 plants, and GFP fluorescence was monitored in the roots. **b** Alternatively, 5(6)-carboxyfluorescein diacetate (CFDA) was applied to grafted Col-0 cotyledons, and fluorescence was monitored in the roots. **c** In addition, fluorescence was monitored in the donor cotyledons after application of CFDA to the recipient roots. **d** Phloem reconnection occurs 3–4 days after grafting as fluorescence was observed in Col-0 roots grafted to *pSUC2::GFP* cotyledons. In addition, Col-0 roots started fluorescing after CFDA application to Col-0 cotyledons in a similar time frame. Xylem reconnection occurs 5–6 days after surgery as grafted cotyledons started fluorescing after CFDA application to the recipient roots. The assays were performed with 3 biological replicates with 10 plants each per day from day 1 to day 7 after grafting at 20 °C and 27 °C. The mean and standard error are shown. Insets show fluorescence in representative grafted plants. Scale bars in insets correspond to 200 μm (root) and 400 μm (cotyledon), respectively

Xylem and phloem reconnection were investigated until 7 days after surgery. To investigate phloem formation, the protocol developed by Melnyk [31] was utilized. Cotyledons of *pSUC2::GFP* seedlings [28] were grafted on Col-0 recipient plants (n = 30) and the roots were monitored daily for the presence of GFP fluorescence (Fig. 3a). The *pSUC2::GFP* line scion expresses free GFP in the phloem which can be transported to sink tissues. The observation of GFP in the wild type root confirms the reconnection of the phloem between scion and rootstock. Alternatively, a vascular tracer, 5(6)-carboxyfluorescein diacetate (CFDA), was applied to identify reconnected phloem or xylem in cot-grafted plants (Fig. 3b, c). For the CFDA assay, the method described by Melnyk et al. [32] was used with the following modifications. For the evaluation of phloem connectivity, 1 µl of a 100 µM CFDA solution was dropped onto the grafted cotyledon. As CFDA has to enter living cells to become fluorescent [33, 34], the leaf was then damaged for better dye penetration using fine forceps. Once CFDA has entered the tissue, it is specifically transported via the vasculature. CFDA was monitored in the roots after 1 h. Rapid shoot-to-root transport of CFDA is typically associated with movement through the phloem [32]. For the evaluation of xylem connectivity, a piece of Parafilm^®^ M was placed directly under the root of the cot-grafted plant. 1 µl of 1 mM CFDA solution was dropped onto the root tip which was then cut for better dye penetration. The transported CFDA was detected in grafted cotyledons after 1 h. Rapid root-to-shoot transport of CFDA is typically associated with movement through the xylem [32]. The fluorescence in all vascular assays was monitored using a stereomicroscope (Nikon SMZ1270) with a GFP-filter. To test a possible temperature effect on cot-grafting (Table 1), the recovery after grafting was conducted at 20 °C or 27 °C.

### Influence of cot-grafting on plant development

**Fig. 4:**
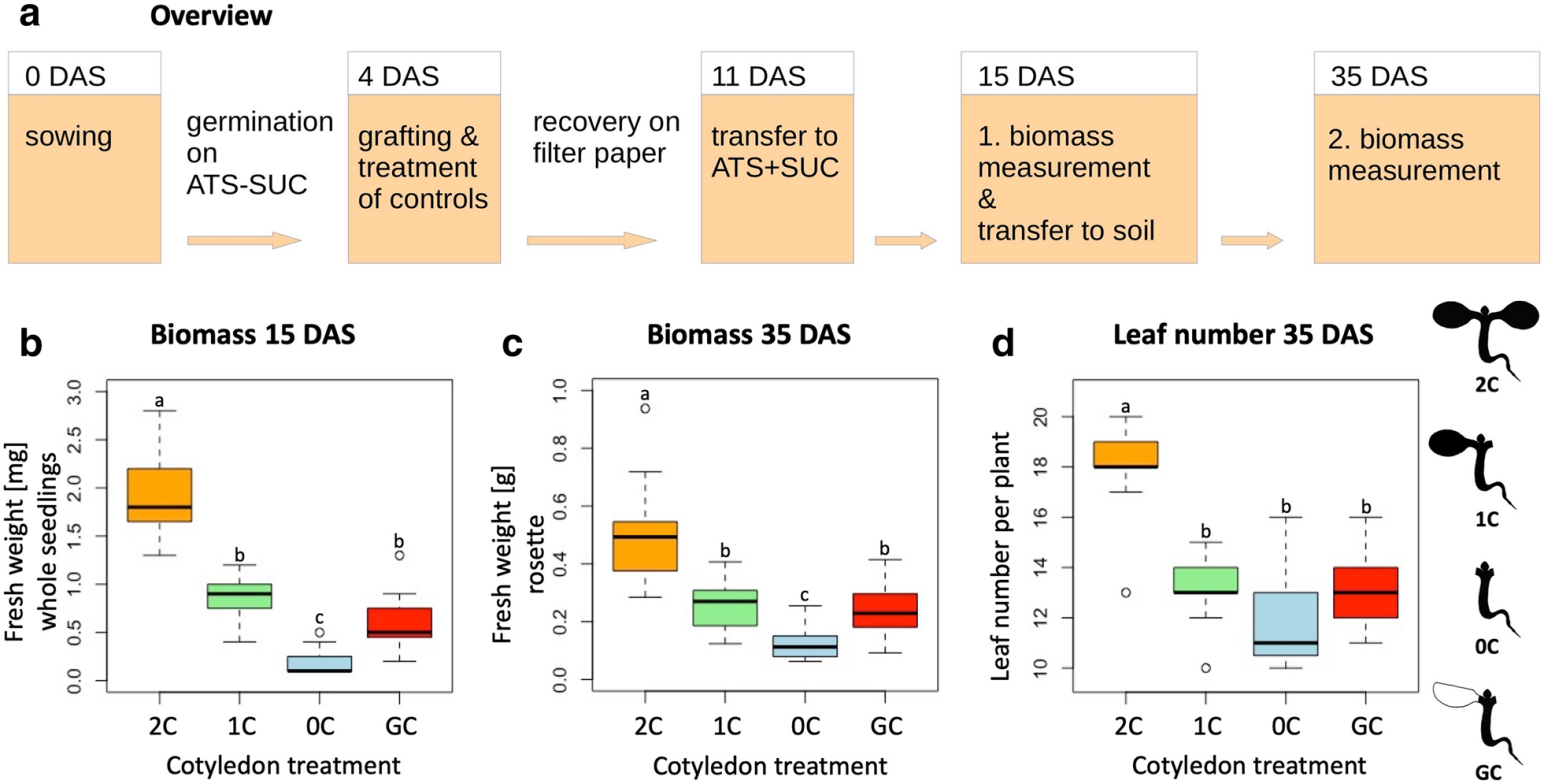
Successful replacement of cotyledons does not result in deficiency in biomass production. The impact of cot-grafting on plant development was investigated by evaluating biomass production at two different developmental stages after grafting. **a** Illustration of the biomass evaluation workflow. **b**–**d** Fresh weight measurements of whole seedlings 15 days after sowing (DAS; **b**). Above ground biomass of single plants 35 DAS (**c**), and number of rosette leaves 35 DAS (**d**). Cot-grafted plants (grafted cotyledon—GC) were compared to three controls: intact plants with two cotyledons (2C), plants without cotyledons (0C) and plants with one cotyledon removed (1C). N = 15; different letters denote statistically significant differences as assessed by 1-way ANOVA followed by a Tukey HSD test (p < .05). SUC, sucrose; ATS, *A. thaliana* solution medium

The impact of the cotyledons on plant development was investigated by comparing biomass production of plants with one grafted cotyledon only and control plants with two, one or without intact cotyledons. For germination and recovery conditions, we used the following combination: 4 days germination in 20 °C on ATS plates without sucrose, short day with a light intensity of 30 µmol * s^−1^ * m^−2^ and 7 days recovery in a vertical position in 20 °C and long days with a light intensity of 90 µmol * s^−1^ * m^−2^. The micrografting procedure was carried out as described above and the cotyledon removal of the control plants was performed under the same conditions. After 7 days of recovery, all plants were transferred to sterile ATS plates containing 1.5% sucrose and kept under recovery conditions for an additional 4 days. For the first biomass measurement 15 days after sowing, the fresh weight of whole seedlings was recorded. Afterwards, the seedlings were transferred to soil and kept in the greenhouse for another 20 days. For the second biomass measurement 35 days after sowing, fresh weight of the above ground material as well as the number of rosette leaves were recorded. The overall workflow is depicted in Fig. 4a.

### Growth conditions for other plant species

Seeds were sown on ATS without sucrose and germinated in 20 °C using short day conditions with a light intensity of 30 µmol * s^−1^ * m^−2^. To allow unfolding of cotyledons, *C. rubella* and *A. suecica* were grafted 5 days after sowing, whereas cotyledon surgery was performed in *B. napus* and *S. lycopersicum* 7 and 11 days after sowing, respectively. The plants recovered in a vertical position in 20 °C, long day with a light intensity of 90 µmol * s^−1^ * m^−2^ for 7 days.

### Statistics

The collected data was statistically assessed by 1-way ANOVA followed by a Tukey HSD test (p < .05).

### Comparing cot-grafting methods

We applied the latest protocol about cot-grafting [13] and compared the results to our method.

## Results and discussion

### Success and limitations of cot-grafting

By applying the established cot-grafting protocol [13], we also achieved a very low success rate of 1.25%, similar to the published rates. To increase the success rates of cot-grafting, we first established hypocotyl micrografting conditions based on [22]. Having achieved success rates over 90% in hypocotyl micrografting, we next developed a new cutting and joining approach for cotyledon micrografting (Figs. 1, 2a) and analyzed different combinations of conditions before and after grafting and their respective success rates (Table 1). The varying factor combinations resulted in cot-grafting success rates ranging from 6 to 92%. Most successful was the combination of short day and low light intensity before grafting and long day, high light intensity, elevated temperature (27 °C) and vertical plate positioning during recovery. This combination led up to 92% success.

Short day conditions were previously reported to enhance the success rate in hypocotyl micrografting [21]. We found that this was also favorable for cot-grafting. Plants grown under low light intensity produce longer hypocotyls and petioles and the plant tissue was much softer. We noticed, however, that despite lower success (up to 62%) the handling of plants grown under high light conditions before grafting was more efficient. The tissue of cotyledons and petioles was stronger and more vigorous under these conditions. Instead of 30 plants per hour using seedlings grown under low light, up to 40 plants could be grafted per hour. Thus, both light intensity conditions lead to a similar output of successfully grafted plants per hour. As such, both growth conditions are justifiable. Moreover, the seedling’s age also has a strong impact on the surgery process. In general, we observed that handling was easier in older and bigger plants. However, the cot-grafting success was not conspicuously influenced by the age of the seedling (Table 1).

Furthermore, as this is manual work, the impact of the experimenter should not be underestimated. While performing hypocotyl micrografting is known to be technically challenging [22], it was observed that cot-grafting is probably even more difficult to perform due to the delicate petioles and cotyledons of young *A. thaliana* seedlings. In our case, success rates between experimenters differed (Table 1). Nevertheless, independent of the experimenter, success rates were consistently high.

Regarding the recovery conditions, elevated temperatures have a dramatic impact on cot-grafting success (Table 1). Although Turnbull et al. [5] and Tsutsui et al. [35] found that high temperatures are helpful for recovery, Melnyk [22] described that high temperatures do not affect total success in hypocotyl micrografting. In contrast, it seems that higher temperatures are more favorable for cot-grafting. According to the recent understanding of thermomorphogenesis [36, 37] in young plant seedlings, high temperatures are perceived in the cotyledons which induces auxin production. Cotyledon-produced auxin is then transported via the petioles to the hypocotyl where it induces brassinosteroid biosynthesis and cellular growth [38]. As a reported key regulator in graft formation [32], additional auxin derived from the cotyledons could promote wound healing. Furthermore, high light intensity and long day conditions seem to provide additional energy via photosynthesis as this has a positive influence on micrografting success after surgery (Table 1). This supplementary energy is probably important for improved wound healing and graft union. In addition, a vertical plate position is advantageous to allow plant growth in its natural orientation.

An example of a successfully grafted *A. thaliana* cotyledon is depicted in Fig. 2b. In addition, well-connected cotyledons were sometimes still present and viable several weeks after grafting (Fig. 2c).

#### Phloem and xylem formation are temporally separated

The chimeric plants with *pSUC2::GFP* cotyledons on Col-0 recipient plants showed reconnecting phloem 3–4 days after grafting. In addition, varying temperatures during recovery caused a dramatic difference in total success rates visible 7 days after grafting (~ 47% at 20 °C vs. ~ 93% at 27 °C). This pattern was also confirmed by CFDA assays (~ 53% at 20 °C vs. ~ 93% at 27 °C; Fig. 3d). Melnyk et al. [32] and Yin et al. [39] reported phloem reconnection in hypocotyl grafted plants 3 days after surgery. Hence, phloem formation in petioles seems to occur in a time frame similar to hypocotyls. CFDA assays confirmed xylem formation 5–6 days after grafting. Melnyk et al. [32] found that xylem is established approximately 7 days after hypocotyl micrografting. Thus, xylem seems to differentiate 1–2 days earlier in petioles than in hypocotyls. Moreover, higher temperatures caused differences in xylem formation as well (7 days after grafting: ~ 43% at 20 °C vs. ~ 87% at 27 °C; Fig. 3e). The observed temperature effects are consistent with our previous success rates (Table 1). Based on these results, the recovery phase should last at least 1 week. Thus, 7 days after grafting is a justifiable time point to assess cot-grafting success.

#### Successful replacement of cotyledons does not result in deficiency in plant growth

As cotyledons seem to have a significant impact on biomass production [25], a successful cot-grafting method must not lead to deficiency in plant growth. Cot-grafted plants should have a comparable development to plants with intact cotyledons. Therefore, we compared biomass production of plants with one grafted cotyledon only to the biomass of control plants with two, one or without intact cotyledons (Fig. 4a).

At the time of the first biomass measurement, 11 days after grafting (15 days after sowing), control plants with two cotyledons had the highest single seedling fresh weight. Plants with one intact cotyledon showed a significantly lower fresh weight which was similar to the fresh weight of plants with one grafted cotyledon (and the other one cut off as shown in Fig. 1h). The lowest fresh weight was measured for control plants without cotyledons (Fig. 4b). This pattern was reproduced for the fresh weight measurements of rosettes 35 days after sowing (Fig. 4c). Only plants with two cotyledons showed a significantly higher leaf number than plants with only one or without cotyledons (Fig. 4d). For all variants we observed that control plants with one intact cotyledon and plants with one grafted cotyledon performed similarly. Consequently, cot-grafting seems to have no major negative influence on cotyledon functionality.

#### Cot-grafting is applicable to other small plant species

**Fig. 5:**
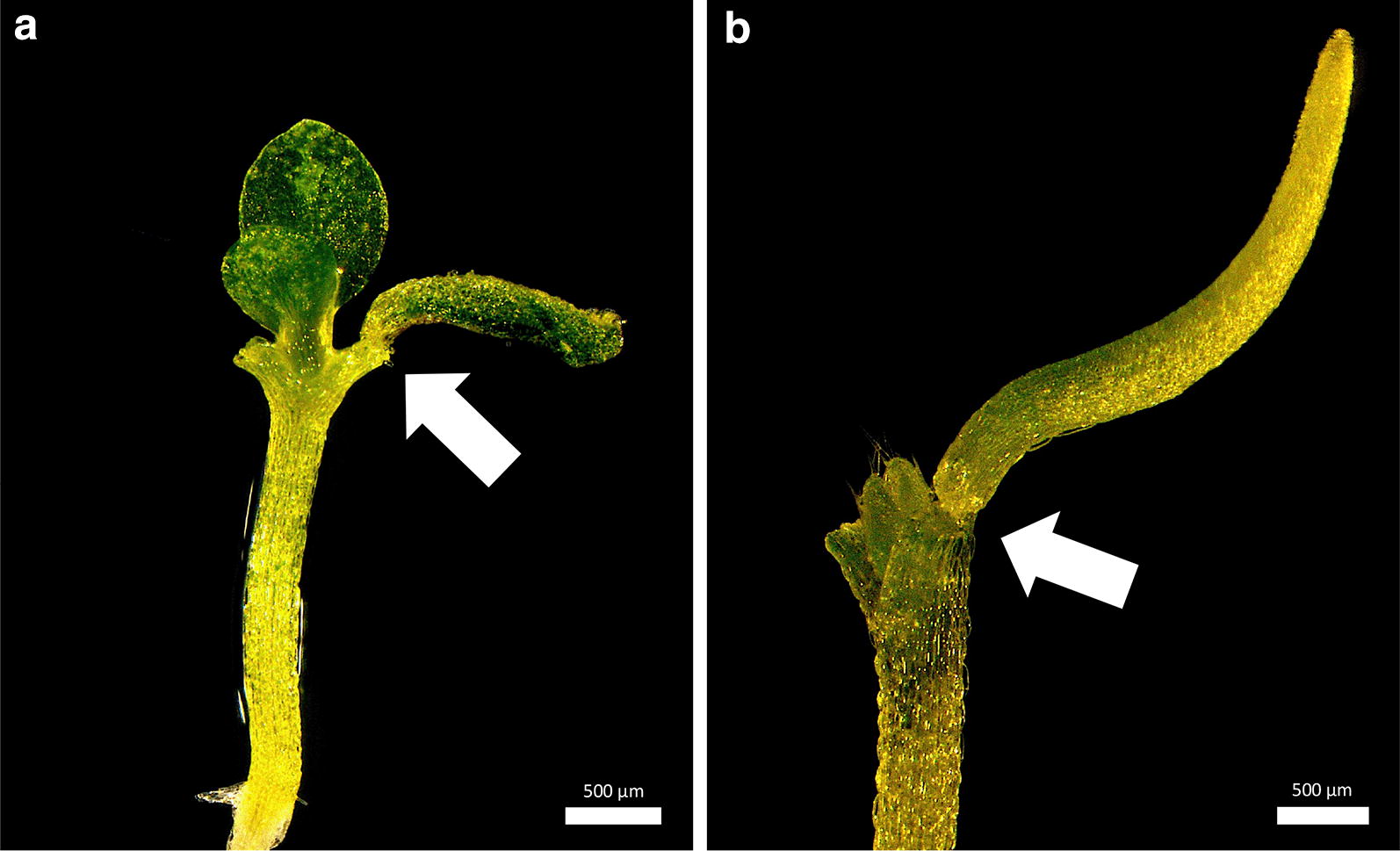
Cotyledon micrografting (cot-grafting) is applicable in related plant species. Seedling of *Arabidopsis suecica* (**a**) and *Capsella rubella* (**b**) with one grafted cotyledon. The seedlings recovered under long day conditions (16 h of light and 8 h of darkness) and 90 µmol * s^−1^ * m^−2^ light intensity and 20 °C for 7 days. The arrows mark graft junctions. Scale bars correspond to 500 μm (**a**, **b**)

For further practical tests, we applied our new cot-grafting method to four other plant species. *Brassica napus* and *Solanum lycopersicum* were chosen to represent larger plants. We could not produce any successfully cot-grafted plants in *Brassica napus* or *Solanum lycopersicum*. One or 2 days after grafting all transplanted cotyledons of these species were detached from the recipients and the hypocotyl had lost contact with the membrane. We hypothesize that the sturdier hypocotyls of *B. napus* and *S. lycopersicum* seedlings nutate more forcefully in their circadian oscillation [12] and, as a consequence, detach easily from the moist surface. In contrast, we successfully grafted *A. suecica* and *C. rubella* as similarly sized relatives of *A. thaliana.* We observed well-connected cotyledons (Fig. 5a, b) with success rates of 8.3% and 5.6% (N = 36), respectively. There is, however, still room for improvement as we used the conditions optimized for *A. thaliana*. Thus, we find that this method is suitable for other small plant species. It could possibly be improved even for larger seedlings by using a stabilizing tube (collar) across the graft junction as previously applied by Turnbull et al. [5] and Nisar et al. [20]. However, the conditions and probably even the procedure need to be adjusted for each species separately.

Recently, cut-in grafting and hypocotyl–hypocotyl flat surface cuttings between *A. thaliana* and *Eutrema salsugineum* were demonstrated by Li et al. [40]. Applying cut and paste cotyledon micrografting also to inter-species combinations would certainly expand the range of questions that could be addressed.

### Scion-to-petiole micrografting

**Fig. 6:**
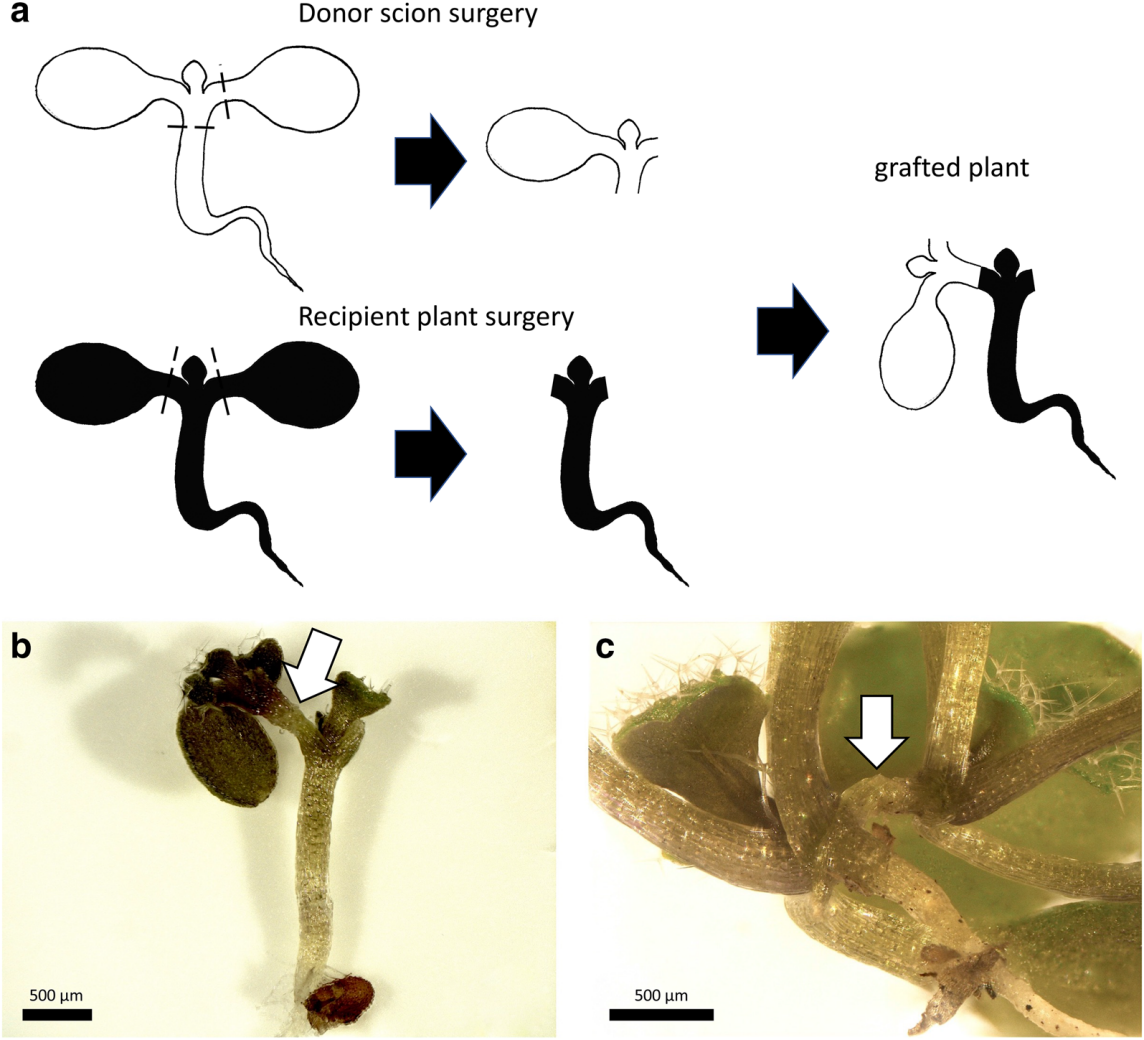
Scion-to-petiole micrografting. **a** The general procedure of scion-to-petiole micrografting: the root and one cotyledon were removed from the donor plant first (donor scion surgery). Both cotyledons of the recipient plant were cut off (recipient plant surgery). The donor scion was then transferred to the recipient plant. Black dashed lines indicate cutting edges. A successfully grafted plant 7 days **b** and 14 days after surgery **c**. The arrows mark graft junctions. Scale bars correspond to 500 μm (**b**, **c**)

Plants are able to reconnect their vasculature between grafted buds and petioles of true leaves which was shown in several plant species [41]. Using the optimized cot-grafting conditions, scions of *A. thaliana* were transplanted from donor plants to petioles of recipient plants as illustrated in Fig. 6a. Plants recovered at 20 °C or 27 °C to investigate the temperature effect. To perform this experiment, 72 plants were grafted for each temperature condition and 54% were well-connected at 20 °C, whereas 71% successfully recovered at 27 °C. Thus, elevated temperatures seem to be advantageous for scion-to-petiole micrografting as well. If grafted scions exhibited adventitious roots (as described by Marsch-Martínez et al. [21]), the plants were counted as unsuccessful grafts. Successfully scion-to-petiole grafted plants are depicted in Fig. 6b, c. These chimeric plants combine two scions from different plants which is similar to the established Y-grafting method in the hypocotyl region [21]. This modification delivers another potentially interesting approach for joining plant organs coming from different origins to study organ-to-organ interactions.

## Conclusions

In general, cot-grafting is a challenging process and requires practice, concentration, and steady hands by the experimenter. A key step in improving success rates from the previously published 2% up to 92% is to perform the micrografting on a flat solid surface instead of culture medium. In addition, adjusted cutting patterns further improved the efficiency of cot-grafting, while high light intensities and elevated temperatures after grafting are beneficial for the formation of the graft union. Similar to hypocotyl micrografting, the phloem connected across the graft junction before the xylem. Regeneration dynamics were rapid, and notably, the cotyledon petiole could regenerate vascular tissues as quickly as or even faster than the hypocotyl, suggesting the cotyledon possesses a strong regenerative capacity despite its short lifespan. The significant impact of cotyledons on biomass and leaf production during early plant development is maintained by successfully grafted cotyledons. Cot-grafting is also possible in other plant species. However, it needs further species-specific adjustment.

## Data Availability

All data generated or analyzed during this study are included in this published article.

## References

[1] Mudge K, Janick J, Scofield S, Goldschmidt EE, Janick J (2009). A history of grafting. Horticultural Reviews.

[2] Lee J-M, Oda M, Janick J (2010). Grafting of herbaceous vegetable and ornamental crops. Horticultural Reviews.

[3] Melnyk CW, Meyerowitz EM (2015). Plant grafting. Curr Biol.

[4] Tsutsui H, Notaguchi M (2017). The use of grafting to study systemic signaling in plants. Plant Cell Physiol.

[5] Turnbull CGN, Booker JP, Leyser HMO (2002). Micrografting techniques for testing long-distance signalling in Arabidopsis. Plant J..

[6] Gasperini D, Chauvin A, Acosta IF, Kurenda A, Stolz S, Chételat A (2015). Axial and radial oxylipin transport. Plant Physiol.

[7] Ragni L, Nieminen K, Pacheco-Villalobos D, Sibout R, Schwechheimer C, Hardtke CS (2011). Mobile gibberellin directly stimulates arabidopsis hypocotyl xylem expansion. Plant Cell.

[8] Matsumoto-Kitano M, Kusumoto T, Tarkowski P, Kinoshita-Tsujimura K, Václavíková K, Miyawaki K (2008). Cytokinins are central regulators of cambial activity. Proc Natl Acad Sci.

[9] Camut L, Regnault T, Sirlin-Josserand M, Sakvarelidze-Achard L, Carrera E, Zumsteg J (2019). Root-derived GA12 contributes to temperature-induced shoot growth in Arabidopsis. Nat. Plants.

[10] Brosnan CA, Mitter N, Christie M, Smith NA, Waterhouse PM, Carroll BJ (2007). Nuclear gene silencing directs reception of long-distance mRNA silencing in Arabidopsis. Proc Natl Acad Sci.

[11] Molnar A, Melnyk CW, Bassett A, Hardcastle TJ, Dunn R, Baulcombe DC (2010). Small silencing RNAs in plants are mobile and direct epigenetic modification in recipient cells. Science.

[12] Takahashi F, Suzuki T, Osakabe Y, Betsuyaku S, Kondo Y, Dohmae N (2018). A small peptide modulates stomatal control via abscisic acid in long-distance signalling. Nature.

[13] Yoo SJ, Hong SM, Jung HS, Ahn JH (2013). The cotyledons produce sufficient FT protein to induce flowering: evidence from cotyledon micrografting in arabidopsis. Plant Cell Physiol.

[14] Green LS, Rogers EE (2004). FRD3 controls iron localization in arabidopsis. Plant Physiol.

[15] Widiez T, Kafafi ESE, Girin T, Berr A, Ruffel S, Krouk G (2011). HIGH NITROGEN INSENSITIVE 9 (HNI9)-mediated systemic repression of root NO_3_—uptake is associated with changes in histone methylation. Proc Natl Acad Sci.

[16] Andersen TG, Nour-Eldin HH, Fuller VL, Olsen CE, Burow M, Halkier BA (2013). Integration of biosynthesis and long-distance transport establish organ-specific glucosinolate profiles in vegetative arabidopsis. Plant Cell.

[17] Chen A, Komives EA, Schroeder JI (2006). An improved grafting technique for mature arabidopsis plants demonstrates long-distance shoot-to-root transport of phytochelatins in arabidopsis. Plant Physiol.

[18] Tsukaya H, Naito S, Rédei GP, Komeda Y (1993). A new class of mutations in Arabidopsis thaliana, acaulis1, affecting the development of both inflorescences and leaves. Development..

[19] Rhee SY, Somerville CR (1995). Flat-surface grafting in *Arabidopsis thaliana*. Plant Mol Biol Report.

[20] Nisar N, Verma S, Pogson BJ, Cazzonelli CI (2012). Inflorescence stem grafting made easy in Arabidopsis. Plant Methods.

[21] Marsch-Martínez N, Franken J, Gonzalez-Aguilera KL, de Folter S, Angenent G, Alvarez-Buylla ER (2013). An efficient flat-surface collar-free grafting method for *Arabidopsis thaliana* seedlings. Plant Methods.

[22] Melnyk CW, Kleine-Vehn J, Sauer M (2017). Grafting with *Arabidopsis thaliana*. Plant hormones: methods and protocols.

[23] Zhu Y, Liu L, Shen L, Yu H (2016). NaKR1 regulates long-distance movement of FLOWERING LOCUS T in *Arabidopsis*. Nat Plants.

[24] Zhang H, Wu Y, Matthew C, Zhou D, Wang P (2008). Contribution of cotyledons to seedling dry weight and development in *Medicago falcata* L. N Z J Agric Res.

[25] Wang L, Liu P-C, Wu LM, Tan J, Peacock WJ, Dennis ES (2019). Cotyledons contribute to plant growth and hybrid vigor in Arabidopsis. Planta.

[26] Penny MG, Moore KG, Lovell PH (1976). The effects of inhibition of cotyledon photosynthesis on seedling development in *Cucumis sativus* L. Ann Bot.

[27] Mugnai S, Azzarello E, Masi E, Pandolfi C, Mancuso S, Mancuso S, Shabala S (2015). Nutation in plants. Rhythms in plants.

[28] Imlau A, Truernit E, Sauer N (1999). Cell-to-cell and long-distance trafficking of the green fluorescent protein in the phloem and symplastic unloading of the protein into sink tissues. Plant Cell.

[29] Podar D, Maathuis FJM (2013). Plant growth and cultivation. Plant mineral nutrients: methods and protocols.

[30] Lincoln C, Britton JH, Estelle M (1990). Growth and development of the axr1 mutants of Arabidopsis. Plant Cell.

[31] Melnyk CW, de Lucas M, Etchhells JP (2017). Monitoring vascular regeneration and xylem connectivity in *Arabidopsis thaliana*. Xylem.

[32] Melnyk CW, Schuster C, Leyser O, Meyerowitz EM (2015). A developmental framework for graft formation and vascular reconnection in *Arabidopsis thaliana*. Curr Biol.

[33] Huang N-C, Yu T-S (2015). A pin-fasten grafting method provides a non-sterile and highly efficient method for grafting Arabidopsis at diverse developmental stages. Plant Methods.

[34] Oparka KJ, Duckett CM, Prior DAM, Fisher DB (1994). Real-time imaging of phloem unloading in the root tip of Arabidopsis. Plant J..

[35] Tsutsui H, Yanagisawa N, Kawakatsu Y, Ikematsu S, Sawai Y, Tabata R, et al. Micrografting device for testing environmental conditions for grafting and systemic signaling in Arabidopsis. bioRxiv 2019;2019.12.20.885525.

[36] Delker C, Sonntag L, James GV, Janitza P, Ibañez C, Ziermann H (2014). The DET1-COP1-hy5 pathway constitutes a multipurpose signaling module regulating plant photomorphogenesis and thermomorphogenesis. Cell Rep..

[37] Quint M, Delker C, Franklin KA, Wigge PA, Halliday KJ, van Zanten M (2016). Molecular and genetic control of plant thermomorphogenesis. Nat Plants.

[38] Bellstaedt J, Trenner J, Lippmann R, Poeschl Y, Zhang X, Friml J (2019). A mobile auxin signal connects temperature sensing in cotyledons with growth responses in hypocotyls. Plant Physiol.

[39] Yin H, Yan B, Sun J, Jia P, Zhang Z, Yan X (2012). Graft-union development: a delicate process that involves cell–cell communication between scion and stock for local auxin accumulation. J Exp Bot.

[40] Li Y, Sun W, Liu F, Cheng J, Zhang X, Zhang H (2019). Methods for grafting *Arabidopsis thaliana* and *Eutrema salsugineum*. Plant Methods.

[41] Wilson PMW (1982). The effect of a grafted bud on vascularization of the petiole in phaseolus, Datura and Lycopersicon. Ann Bot.

